# *Agrobacterium*-Mediated Genetic Transformation of Wild *Oryza* Species Using Immature Embryos

**DOI:** 10.1186/s12284-020-00394-4

**Published:** 2020-06-03

**Authors:** Sae Shimizu-Sato, Katsutoshi Tsuda, Misuzu Nosaka-Takahashi, Toshiya Suzuki, Seijiro Ono, Kim Nhung Ta, Yuri Yoshida, Ken-Ichi Nonomura, Yutaka Sato

**Affiliations:** National Institute of Genetics, 1111 Yata, Mishima, Shizuoka, 411-8540 Japan

**Keywords:** *Agrobacterium*-mediated transformation, Immature embryo, *Oryza*, Wild accession

## Abstract

Genetic transformation is one of the most important technologies for revealing or modulating gene function. It is used widely in both functional genomics and molecular breeding of rice. Demands on its use in wild *Oryza* species is increasing because of their high genetic diversity. Given the difficulties in genetic crosses between distantly related species, genetic transformation offers a way to alter or transfer genetic traits in wild rice accessions. However, transformation of wild *Oryza* accessions by conventional methods using calli induced from scutellum tissue of embryos in mature seeds often fails. Here, we report methods using immature embryos for the genetic transformation of a broad range of *Oryza* species. First, we investigated the ability of callus induction and regeneration from immature embryos of 192 accessions in 20 species under several culture conditions. We regenerated plants from immature embryos of 90 accessions in 16 species. Next, we optimized the conditions of *Agrobacterium* infection using a vector carrying the *GFP* gene driven by the maize ubiquitin promoter. GFP signals were observed in 51 accessions in 11 species. We analyzed the growth and seed set of transgenic plants of *O. barthii*, *O. glumaepatula*, *O. rufipogon*, and *O. brachyantha*. The plants grew to maturity and set seeds normally. Southern blot analyses using DNA from T_0_ plants showed that all GFP plants were derived from independent transformation events. We confirmed that the T-DNAs were transmitted to the next generation through the segregation of GFP signals in the T_1_ generation. These results show that many *Oryza* species can be transformed by using modified immature-embryo methods. This will accelerate the use of wild *Oryza* accessions in molecular genetic analyses and molecular breeding.

## Background

Rice (*Oryza sativa* L.) is one of the most important crops in the world. Breeding new rice cultivars with strong tolerance to environmental changes caused by global warming is an urgent goal. Because wild *Oryza* species have high genetic diversity and thus have better chances of being adapted to fluctuating environments, their use in basic science and breeding is increasing (Morishima and Oka 1970; Vaughan 1994). Wild *Oryza* accessions have been studied to analyze their drought tolerance (Zhang et al. 2006), shattering (Ishii et al. 2013), awn formation (Ikemoto et al. 2017), zinc accumulation (Ishikawa et al. 2017), salt tolerance (Yichie et al. 2018), yield (Fan et al. 2019), and disease resistance (Kariya et al. 2019). These studies identified genes regulating potential agriculturally important traits. The accumulation of whole genome sequences of wild *Oryza* species (Oryzabase, https://shigen.nig.ac.jp/rice/oryzabase/; Gramene, http://www.gramene.org) provides opportunities in both basic and applied studies (Stein et al. 2018; Zhao et al. 2018; Shenton et al. 2020).

Genetic transformation is one of the most important technologies for revealing or modulating gene function, and is often used in functional genomics and molecular breeding. Several methods for transferring genes into rice (Shimamoto et al. 1989) have been developed, including particle bombardment (Toriyama et al. 1988), polyethylene glycol-mediated (Zhang and Wu 1988), and *Agrobacterium*-mediated methods (Potrykus 1991; Hiei et al. 1994). Through the improvement of *Agrobacterium*-mediated methods, transformation efficiency has become high, and transgenes with single or low copy numbers or large DNA segments with defined ends can be integrated into host genomes (Hiei et al. 1994). *Agrobacterium*-mediated transformation in rice uses two main types of starting materials: calli induced from scutellum tissue of embryos in mature seeds, and immature embryos at approximately 10 days after fertilization. The former method is more convenient because we can collect and stock enough mature seeds to run experiments at any time. The latter method can be applied to a much wider range of cultivated rice (Hiei and Komari 2008) and is applicable to various crop species, including maize (Ishida et al. 1996), wheat (Ishida et al. 2005), and sorghum (Sato-Izawa et al. 2018). Thus, the immature embryo method may be applicable to wild *Oryza* species, for which the mature embryo method is often unsuccessful.

*Oryza* comprises two cultivated species (*O. sativa* and *O. glaberrima*) and 21 wild species (Vaughan 1989, 1994) with 9 genome types—AA, BB, CC, BBCC, CCDD, EE, FF, GG, and HHJJ—according to cytogenetic observations of interspecific F_1_ hybrids and total genomic DNA hybridization (Khush et al. 2001). The two cultivated species have the AA genome. The introduction of agronomically useful genes from wild accessions into cultivated species is highly valuable. However, the introduction of those genes from wild accessions with the AA genome into cultivated species by genetic crosses followed by repeated backcrosses is time consuming and labor intensive. In addition, genes linked to genes for reproductive barriers or for undesirable characters are difficult to introduce alone by crossing. The introduction of genes from wild accessions with other genomes by genetic crosses is even harder. Usually, crosses between different genome types do not produce stable offspring. In some cases, aneuploid plants can be obtained but their chromosome composition is unstable and not healthy. Genetic transformation offers promise in the transfer of genes from wild accessions, especially with genomes other than AA, and allows the functional analysis of genes in wild *Oryza* accessions. The Japanese National Institute of Genetics (NIG) holds in Oryzabase and distributes more than 1700 accessions of wild *Oryza* species. Here, we used the immature embryo method for the genetic transformation of wild rice accessions from a broad range of species with various media and *Agrobacterium* infection conditions and show that many *Oryza* species can be transformed by the immature embryo method.

## Materials and Methods

### Plant Materials

Seeds of wild *Oryza* species were supplied by NIG. Plants were grown under natural light in a greenhouse and transplanted into a paddy field in June in Mishima, Japan. At 1 month after transplanting, daylength was controlled 12.5 h light / 11.5 h dark to induce flowering. *Oryza sativa* ‘Nipponbare’ was grown as a control in similar conditions but under natural daylength. At 8 to 10 days after flowering, immature embryos were isolated.

### Plant Regeneration from Immature Embryos

Regeneration tests using immature embryos were conducted according to Hiei and Komari (2008). All culture media are detailed in Supplemental Table 1. Ovaries were sterilized in 70% ethanol (v/v) for 1 min, followed by 15 min in 50% (v/v) sodium hypochlorite solution with shaking, and washed 5 times in sterile distilled water. Hiei and Komari (2008) used two types of callus-inducing media, nN6C and CCMC, to induce calli from *japonica* and *indica* cultivars, respectively. The immature embryos were isolated under a stereomicroscope on a clean bench, placed on nN6C or CCMC medium containing 25 mg/L meropenem, and cultured at 32 °C for 7 days. Then they were transferred to the same fresh media and cultured for another 7 days under the same conditions. Next, we tested regeneration of shoots and roots in four combinations of media, designated A to D (Supplemental Table 2). Calli formed on the immature embryos were transferred to either regeneration medium (N6R) or pre-regeneration medium (NBPRC). On N6R, calli were cultured at 32 °C for 2 weeks, and regenerated plantlets were transferred to rooting medium (N6F). On NBPRC, calli were cultured at 32 °C for 1 week, and were then transferred to regeneration medium (RNM). After 2 weeks on RNM, regenerated plantlets were transferred to rooting medium (MSI). Plants which formed roots on N6F or MSI were eventually transferred to soil in pots and grown to maturity in the greenhouse.

### Binary Vectors and Transformation of *Agrobacterium tumefaciens*

The binary plasmid vector (pPUG1–1) used in this study contains a *hygromycin phosphotransferase* (*hpt*) gene under the control of the cauliflower mosaic virus 35S promoter, and a *green fluorescent protein* (*GFP*) gene under the control of the maize ubiquitin promoter. The vector was transferred into *Agrobacterium tumefaciens* strains LAB4404 and EHA105 by electroporation in an *E. coli* pulser (Bio-Rad).

### *Agrobacterium*-Mediated Transformation

*Agrobacterium*-mediated transformation was conducted according to Hiei and Komari (2008). All media included 25 mg/L meropenem instead of cefotaxime and carbenicillin. Strain LBA4404 or EHA105 harboring pPUG1–1 was grown on LB medium supplemented with 50 mg/L hygromycin at 28 °C for 3 days. The immature embryos were held in a water bath at 43 °C for 30 min, and then cooled in water. They were then centrifuged in an angle rotor at either 1100× or 20,000×*g* for 10 min at 22 °C. They were placed on co-cultivation medium containing 100 μM acetosyringone with the scutellum side up, and 5 μL of *Agrobacterium* suspension was dropped on them. After incubation for 7 days at 25 °C in the dark, plants were regenerated as described in the previous section, with the addition of hygromycin into all media for selection.

### Observation of GFP Fluorescence

GFP fluorescence was observed under a fluorescence microscope with a GFP filter (SZX16, Olympus).

### Extraction of Rice Genomic DNA and Southern Blot Analysis

Total genomic DNA was extracted from ca. 1 g of fresh leaf of each transgenic line with a DNeasy Plant Maxi Kit (Qiagen) according to the manufacturer’s protocol. PCR analysis to detect the full-length *hpt* gene was performed using GoTaq DNA polymerase (Promega) with the primer set 5′-ATGAAAAAGCCTGAACTCACCGCG-3′ / 5′-CTATTCCTTTGCCCTCGGACGAGT-3′.

Southern blot analysis was performed with an ECL Direct Nucleic Acid Labelling and Detection System (GE Healthcare) according to the manufacturer’s protocol. Genomic DNA (5 μg) was digested by *Hin*dIII (Takara) and separated by electrophoresis. For the preparation of *hpt* probe, the full-length *hpt* gene was amplified with the primers used for PCR analysis above. Southern blot hybridization signals were detected on an Image Quant LAS 4000 Mini imager (GE Healthcare).

## Results and Discussion

### Optimization of Culture Media for Regeneration of Plantlets from Immature Embryos from Wild *Oryza* Accessions

First we tested the response of immature embryos to callus induction. From 192 accessions in 20 wild *Oryza* species, 138 accessions in 19 species formed callus on either or both media (Table 1). Using these 138 accessions, we tested the regeneration of shoots and roots in conditions A and B, and then using accessions which did not respond to either, in conditions C and D. Twenty-eight accessions regenerated from immature embryo calli in conditions A and B, 18 only in condition A, and 38 only in condition B (Table 2). Of 54 accessions which did not respond to A or B, 4 regenerated under C and 2 under D (Table 2).

**Table 1:** Callus induction from immature embryos of wild *Oryza* accessions on different callus-inducing media

Type of medium	nN6C, CCMC	nN6C	CCMC	No callus induced on either
*O. barthii*, AA	W1473, W1583	W1467, W1605, W1643	W0747, W1588, W1702	W1063, W1416
*O. glumaepatula*, AA	W1189, W1191, W2165, W2184, W2203	W1167, W1169, W1477, W2145, W2160, W2201	W1183, W1185, W2140, W2149, W2173, W2192	W1187
*O. longistaminata*, AA	W1573			
*O. meridionalis*, AA	W1635, W2100, W2112	W1297, W1300, W1631, W1638	W1625, W2071, W2077, W2080, W2081, W2105	W2079, W2103
*O. rufipogon*, AA	W0107, W0593, W0625, W1230, W1239, W1294, W1551, W1685, W1852, W1865, W1965, W2014, W2265	W0629, W0630, W1235, W1739, W1807, W1866, W1944, W1962, W2003, W2051	W0102, W0106, W0137, W0610, W0621, W1114, W1666, W1669, W1681, W1690, W1718, W1921, W1945, W2078, W2109, W2263	W0120, W0128, W1802, W1825, W2050
*O. punctata* (2*x*), BB	W1515, W1582, W1586, W1593	W1514, W1577, W1590, W1592		
*O. eichingeri*, CC		W1525		W1526
*O. officinalis*, CC	W1315	W1291	W0002, W0614, W1301, W1830, W1930	W0065, W0566, W1131, W1200, W1252, W1302, W1308, W1361, W1814
*O. rhizomatis*, CC		W1805, W1812	W1808	
*O. minuta*, BBCC	W1323	W0016, W1213	W0051, W1318, W1319, W1329, W1331, W1336, W1342	W0045, W1328
*O. punctata* (4*x*), BBCC	W1474(B)		W1024, W1408, W1564	W0043, W1023, W1145, W1409
*O. alta*, CCDD		W0017, W0018		W1147
*O. grandiglumis,* CCDD	W1483	W1480(B), W2220	W0613, W1195, W1476	W1247
*O. latifolia*, CCDD	W1177, W1197	W0047, W1539		W0048, W0542, W1166, W1168, W1181, W1184
*O. australiensis*, EE	W0008, W1639	W1632, W2082, W2084		W1296, W1628, W1630, W2086, W2104
*O. brachyantha*, FF	W1401	W0654	W0656, W1407(B), W1706, W1711	W1403
*O. granulata*, GG	W0003		W0067(B)	W0004, W0615
*O. meyeriana*, GG	W2068			W1348
*O. longiglumis*, HHJJ	W1227			W1218, W1219, W1220, W1222, W1223, W1224, W1228, W1229
*O. ridleyi*, HHJJ				W0001, W2033, W2035
Sub-subtotal	40	43	55	54
Subtotal	138			
Total	192

**Table 2:** Regeneration from immature embryos of wild *Oryza* accessions on 4 medium combinations (A–D)

Species, genome type	Accession	A and B	A alone	B alone	C	D
*O. barthii*, AA	W0747		0/8	4/16		
	W1467		5/8	0/8		
	W1473	4/16				
	W1583	6/16				
	W1588		0/8	3/8		
	W1643		5/8	0/8		
	W1702		0/8	6/8		
*O. glumaepatula*, AA	W1183		0/8	6/8		
	W1189	5/16				
	W1191	10/16				
	W1477		0/8	0/8	4/8	0/8
	W2140		0/8	6/8		
	W2145		0/8	0/8	2/8	0/7
	W2149		0/8	8/16		
	W2160		0/8	0/8	3/8	0/8
	W2165	13/16				
	W2173		0/8	10/16		
	W2184	12/16				
	W2192		0/8	5/8		
	W2201		0/7	0/8	2/8	0/7
	W2203	9/16				
*O. longistaminata*, AA	W1573	2/40				
*O. meridionalis*, AA	W1300		2/16	0/8		
	W1625		0/8	3/24		
	W1631		2/12	0/8		
	W1635	3/16				
	W2071		0/8	1/16		
	W2080		0/8	1/16		
	W2105		0/8	2/16		
*O. rufipogon*, AA	W0102		0/8	4/8		
	W0106		0/8	3/8		
	W0107	12/16				
	W0137		0/8	7/16		
	W0593	10/16				
	W0610		0/8	6/16		
	W0621		0/7	2/8		
	W0625	7/16				
	W0629		3/8	0/8		
	W1230	2/16				
	W1239	11/16				
	W1551	13/16				
	W1666		0/8	5/9		
	W1669		0/8	6/8		
	W1681		0/8	4/8		
	W1685	12/16				
	W1690		0/8	4/8		
	W1718		0/8	4/8		
	W1739		3/8	0/7		
	W1852	5/16				
	W1865	12/16				
	W1944		3/8	0/8		
	W1945		0/8	4/8		
	W1962		3/8	0/8		
	W2014	11/16				
	W2051		4/8	0/8		
	W2263		0/8	5/9		
	W2265	13/16				
*O. punctata* (2*x*), BB	W1582	5/16				
	W1586	4/16				
	W1590		3/8	0/8		
	W1592		2/8	0/8		
	W1593	4/16				
*O. eichingeri*, CC	W1525		2/24	0/16		
*O. officinalis*, CC	W0614		0/8	2/15		
	W1301		0/8	3/16		
	W1830		0/7	1/16		
	W1930		0/8	2/15		
*O. rhizomatis*, CC	W1808		0/8	3/16		
	W1812		4/16	0/16		
*O. minuta*, BBCC	W0051		0/8	3/16		
	W1213		2/8	0/8		
	W1318		0/8	0/8	0/8	1/8
	W1323	3/15				
	W1331		0/8	0/8	0/8	1/8
*O. punctata* (4*x*), BBCC	W1474(B)	2/16				
	W1564		0/16	2/16		
*O. alta*, CCDD	W0017		2/16	0/12		
*O. grandiglumis,* CCDD	W0613		0/8	4/8		
	W1195		0/8	3/16		
	W1476		0/8	4/16		
	W1480(B)		3/16	0/8		
	W1483	6/16				
	W2220		4/16	0/8		
*O. latifolia*, CCDD	W1177	2/16				
	W1539		1/24	0/16		
*O. brachyantha*, FF	W0656		0/8	5/8		
	W1407(B)		0/8	4/8		
	W1706		0/8	3/8		
	W1711		0/8	5/8		
*O. longiglumis*, HHJJ	W1227	2/32				
Subtotal		28	18	38	4	2
Total		90				

number of regenerated plants / total number of tested immature embryos

Overall, among the 192 accessions tested, 90 formed calli from immature embryos and regenerated in tissue culture under one or more of the conditions tested. These accessions include AA, BB, CC, BBCC, CCDD, FF, and HHJJ genome species. Thus, we have identified conditions of tissue culture and the recovery of plantlets suitable for a wide range of *Oryza* species except those with EE and GG genomes. Condition B covered the widest range of wild *Oryza* accessions, followed by condition A. Very few of the accessions that did not respond to condition A or B responded to condition C or D. In particular, 4 accessions of *O. glumaepatula* (W1477, W2145, W2160, and W2201) responded to condition C, while other accessions of the same species responded to A or B. Thus, each species may have a genotype-dependent optimal condition for the regeneration of plantlets from immature embryos.

In general, species with the AA genome had a high regeneration success rate, notably *O. barthii*, *O. glumaepatula*, *O. meridionalis*, and *O. rufipogon* (Table 3). Although the success rate in *O. longistaminata* was 100%, we tested only one accession. As *O. longistaminata* generally propagates vegetatively through rhizomes rather than sexually, the immature embryo method is not particularly applicable for its transformation. We could not regenerate plantlets from immature embryos of *O. australiensis*, *O. granulata*, *O. meyeriana*, or *O. ridleyi*. Of 10 *O. australiensis* (EE) accessions tested, 5 formed calli and green spots but did not form shoots. Of 4 accessions of *O. granulata* and 2 of *O. meyeriana* (both GG) tested, 2 and 1 accessions, respectively, produced calli but did not form green spots or regenerate shoots and roots. It is well known that the plant hormone cytokinin plays an important role in shoot regeneration from calli. Further consideration of medium conditions, such as the concentration of cytokinin, may be necessary to induce regeneration from immature embryos of wild *Oryza* species with EE and GG genomes.

**Table 3:** Summary of regeneration tests from immature embryos of wild *Oryza* species

Species, genome type	No. accessions tested	No. accessions with callus induction^a^	No. accessions with regeneration^b^	Percentage of regenerated accessions^c^
*O. barthii*, AA	10	8 (80%)	7 (88%)	70%
*O. glumaepatula*, AA	18	17 (94%)	14 (82%)	78%
*O. longistaminata*, AA	1	1 (100%)	1 (100%)	100%
*O. meridionalis*, AA	15	13 (87%)	7 (54%)	47%
*O. rufipogon*, AA	44	39 (89%)	28 (72%)	64%
*O. punctata* (2*x*), BB	8	8 (100%)	5 (63%)	63%
*O. eichingeri*, CC	2	1 (50%)	1 (100%)	50%
*O. officinalis*, CC	16	7 (44%)	4 (57%)	25%
*O. rhizomatis*, CC	3	3 (100%)	2 (67%)	67%
*O. minuta*, BBCC	12	10 (83%)	5 (50%)	42%
*O. punctata* (4*x*), BBCC	8	4 (50%)	2 (50%)	25%
*O. alta*, CCDD	3	2 (67%)	1 (50%)	33%
*O. grandiglumis*, CCDD	7	6 (86%)	6 (100%)	86%
*O. latifolia*, CCDD	10	4 (40%)	2 (50%)	20%
*O. australiensis*, EE	10	5 (50%)	0 (0%)	0%
*O. brachyantha*, FF	7	6 (86%)	4 (67%)	57%
*O. granulata*, GG	4	2 (50%)	0 (0%)	0%
*O. meyeriana*, GG	2	1 (50%)	0 (0%)	0%
*O. longiglumis*, HHJJ	9	1 (11%)	1 (100%)	11%
*O. ridleyi*, HHJJ	3	0 (0%)	0 (0%)	0%
Total	192	138 (72%)	90 (65%)	47%

^a^100 × number of accessions with induced calli / total number of tested accessions

^b^100 × number of regenerated accessions / number of accessions with induced calli

^c^100 × number of regenerated accessions / total number of tested accessions

### Optimization of *Agrobacterium* Infection of Immature Embryo Calli from Wild *Oryza* Accessions

**Fig. 1:**
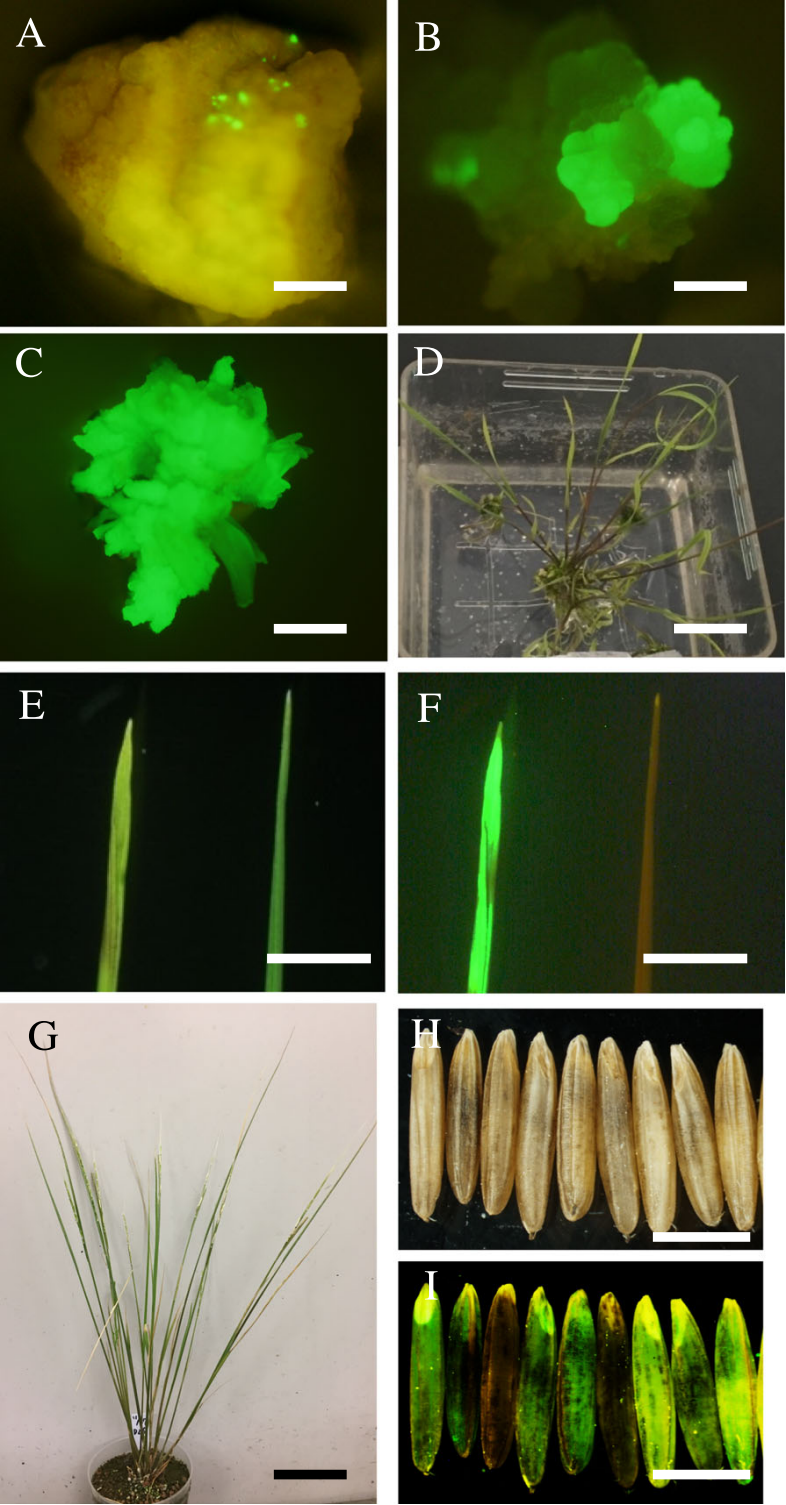
*Agrobacterium-*mediated transformation of *Oryza brachyantha* W1711. **a** At 10 days after infection, GFP signals were observed in immature embryos. Bar = 1 mm. **b** Callus was cultured for 2 weeks on selection medium. Part of the callus showed a GFP signal. Bar = 2 mm. **c** After 2 weeks’ culture on regeneration media, shoots with strong GFP signals were formed. Bar = 3 mm. **d** Regenerated plants on rooting medium. Bar = 2 cm. **e**, **f** Leaves through **e** bright field and **f** GFP filters: left, transgenic; right, non-transgenic. Bar = 1 cm. **g** Transgenic plants growing to maturity without morphological abnormalities. Bar = 5 cm. **h**, **i** T_1_ seeds of a transgenic plant through **h** bright field and **i** GFP filters. T_1_ seeds with and without GFP signal were segregated. Bar = 4 mm

Next, we compared the conditions of *Agrobacterium* infection of calli. Heat treatment and centrifugation of calli before *Agrobacterium* infection can increase the transformation efficiency in cultivated rice, maize, wheat, and sorghum (Hiei et al. 2006; Ishida et al. 2014; Sato-Izawa et al. 2018). Transformation efficiency is also affected by *Agrobacterium* strains (Hiei and Komari 2008). Therefore, we tested heat treatment, two conditions of centrifugation, and two *Agrobacterium* strains (Table 4). To monitor infection, we used a vector carrying the *GFP* driven by the ubiquitin promoter. Following infection, we counted the number of immature embryos with GFP-positive spots at 10 days (Fig. 1a). Using calli induced on immature embryos of Nipponbare, we showed that neither the speed of centrifugation nor the *Agrobacterium* strain affected the efficiency of infection (Table 4), possibly owing to very high infection efficiency in all conditions tested. Next, we infected calli of 16 wild *Oryza* accessions, one per species in which regeneration was achieved, and measured infection (Table 4). As wild accessions tend to flower gradually in a panicle, it is difficult to collect many ovaries at 8 to 10 day after pollination (DAP) at the same time, so we used 8 to 25 immature embryos of one accession of each species. GFP fluorescence showed that 11 out of 16 accessions were infected in one or more conditions tested (Table 4). This rate is considerably lower than that of Nipponbare. Nipponbare panicles flower in a brief period, so one can collect many immature embryos at once at the ideal stage for *Agrobacterium* infection. This difference could be a reason for the high infection ratio in Nipponbare. Another possibility is that the developmental window of immature embryos which can accept *Agrobacterium* infection may be broader in Nipponbare than in wild accessions.

**Table 4:** Efficiency of *Agrobacterium* infection of calli induced from immature embryos of 16 wild *Oryza* species

	Strain Centrifugation	LBA4404		EHA105	
		1100×*g*	20,000×*g*	1100×*g*	20,000×*g*
1	*O. barthii*, W1467, AA	1/9	2/8	0/8	1/8
2	*O. glumaepatula*, W2184, AA	3/8	3/9	1/8	2/8
3	*O. longistaminata*, W1573, AA	0/8	0/8	0/8	1/8
4	*O. meridionalis*, W2080, AA	0/9	1/8	0/8	0/8
5	*O. rufipogon*, W1551, AA	3/8	2/8	0/8	1/8
6	*O. punctata* 2*x*, W1582, BB	2/8	0/8	0/8	0/8
7	*O. eichingeri*, W1525, CC	0/8	0/8	1/8	0/8
8	*O. officinalis*, W1301, CC	0/16	0/16	0/17	0/16
9	*O. rhizomatis*, W1808, CC	0/16	0/15	0/16	0/17
10	*O. minuta*, W0051, BBCC	0/8	0/8	1/8	0/8
11	*O. punctata* 4*x*, W1564, BBCC	0/8	0/8	1/8	0/8
12	*O. alta*, W0017, CCDD	1/8	1/8	0/8	0/8
13	*O. grandiglumis*, W2220, CCDD	0/16	0/18	0/16	0/18
14	*O. latifolia*, W1177, CCDD	0/24	0/25	0/24	0/24
15	*O. brachyantha*, W1711, FF	2/9	1/8	0/8	1/8
16	*O. longiglumis*, W1227, HHJJ	0/24	0/24	0/25	0/24
17	*O. sativa*, Nipponbare, AA	88/90	76/79	77/83	81/85

number of GFP positive calli / total number of tested immature embryos

Next, we tested *Agrobacterium* infection in 60 more wild accessions (Table 5), mainly under conditions in which an accession of the same species was infected (Table 4). Of the 76 accessions in 16 species, infection was successful in 51 accessions in 11 species. In most species (*O. barthii*, *O. glumaepatula*, *O. longistaminata*, *O. rufipogon*, *O. punctata* (2*x*), *O. eichingeri*, *O. minuta*, *O. punctata* (4*x*), *O. alta*, and *O. brachyantha*), infection and GFP fluorescence had a relatively high success rate (≥50%). However, in *O. meridionalis*, only 1 among 7 accessions showed a GFP signal. This result suggests that this species may have a mechanism that suppresses *Agrobacterium* infection in these conditions. Infection was not successful in *O. officinalis*, *O. rhizomatis*, *O. grandiglumis*, *O. latifolia*, or *O. longiglumis*. However, only 1 or 2 accessions were used for these species, so it is not clear whether this is a general tendency in each species. Further consideration of the conditions of infection, such as cold shock treatment, may be necessary (Sato-Izawa et al. 2018).

**Table 5:** Efficiency of *Agrobacterium* infection of calli induced from immature embryos of 76 accessions of wild *Oryza*

Species, genome type	Strain	LBA4404		EHA105		Infection efficiency^a^
	Centrifugation	1100×*g*	20,000×*g*	1100×*g*	20,000×*g*	
*O. barthii*, AA	W1467^b^	1/9	2/8	0/8	1/8	3/6 (50%)
	W1473	0/8	0/8			
	W1583				1/8	
	W1588		0/8		0/8	
	W1643		0/8		0/8	
	W1702		2/9			
*O. glumaepatula*, AA	W1183		1/8			13/14 (93%)
	W1189	0/8	0/8			
	W1191		2/8			
	W1477	1/8	3/16			
	W2140	1/8	1/8			
	W2145		1/8			
	W2149	2/8				
	W2160		2/8		3/16	
	W2165	1/8				
	W2173		3/16		2/16	
	W2184^b^	3/8	3/9	1/8	2/8	
	W2192	2/8				
	W2201	1/8	1/8			
	W2203	1/8	2/8			
*O. longistaminata*, AA	W1573^b^	0/8	0/8	0/8	1/8	1/1 (100%)
*O. meridionalis*, AA	W1300	0/9	0/8			1/7 (14%)
	W1625		0/8	0/8		
	W1631		0/8	0/8		
	W1635	0/8	0/7			
	W2071	0/8	0/8			
	W2080^b^	0/9	1/8	0/8	0/8	
	W2105		0/8	0/8		
*O. rufipogon*, AA	W0102		1/8			21/26 (81%)
	W0106	1/8				
	W0107	2/8	2/16			
	W0137	0/8	0/8			
	W0593		1/8			
	W0610		1/8		1/8	
	W0621	1/8	1/8			
	W0625	1/9	1/8			
	W0629	1/8	1/8			
	W1230	0/8	0/8			
	W1239	2/8				
	W1551^b^	3/8	2/8	0/8	1/8	
	W1666	1/8				
	W1669	2/8				
	W1681	1/8				
	W1685	2/8	2/8			
	W1718	0/8	0/8			
	W1739	0/8	0/8			
	W1852		1/8			
	W1865	2/8	1/8			
	W1945		1/8			
	W1962		0/8		0/8	
	W2014	2/8			1/8	
	W2051		1/8			
	W2263		1/8			
	W2265	1/8	2/8			
*O. punctata* (2*x*), BB	W1582^b^	2/8	0/8	0/8	0/8	3/4 (75%)
	W1586	1/16				
	W1592	0/8	0/8			
	W1593	1/16				
*O. eichingeri*, CC	W1525^b^	0/8	0/8	1/8	0/8	1/1 (100%)
*O. officinalis*, CC	W1301^b^	0/16	0/16	0/17	0/16	0/2 (0%)
	W1830	0/8	0/8			
*O. rhizomatis*, CC	W1808^b^	0/16	0/15	0/16	0/17	0/2 (0%)
	W1812		0/8	0/8		
*O. minuta*, BBCC	W0051^b^	0/8	0/8	1/8	0/8	2/3 (67%)
	W1323				1/8	
	W1331			0/8	0/8	
*O. punctata* (4*x*), BBCC	W1474(B)			1/8		2/2 (100%)
	W1564^b^	0/8	0/8	1/8	0/8	
*O. alta*, CCDD	W0017^b^	1/8	1/8	0/8	0/8	1/1 (100%)
*O. grandiglumis*, CCDD	W2220^b^	0/16	0/18	0/16	0/18	0/1 (0%)
*O. latifoli*a, CCDD	W1177^b^	0/24	0/25	0/24	0/24	0/1 (0%)
*O. brachyantha*, FF	W0656	2/8	2/8		2/8	3/4 (75%)
	W1407(B)	1/8				
	W1706	0/8			0/8	
	W1711^b^	2/9	1/8	0/8	1/8	
*O. longiglumis*, HHJJ	W1227^b^	0/24	0/24	0/25	0/24	0/1 (0%)
Total						51/76 (67%)

number of GFP positive calli / total number of tested immature embryo

^a^100 × number of GFP positive accessions / total number of tested accessions

^b^same data as Table 4

### Generation of Transgenic Plants and Inheritance of Transgene in Wild *Oryza* Accessions

**Fig. 2:**
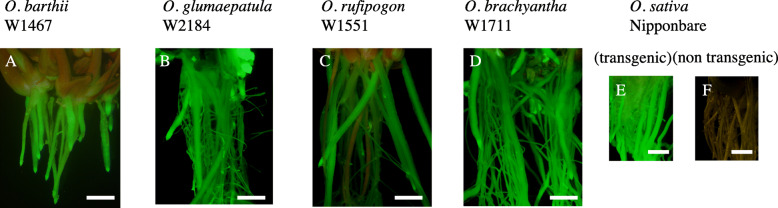
*Agrobacterium-*mediated transformation of wild *Oryza*. **a**–**e**) GFP fluorescence in roots of transgenic wild rice: **a***O. barthii* W1467, **b***O. glumaepatula* W2184, **c***O. rufipogon* W1551, **d***O. brachyantha* W1711, and **e***O. sativa* Nipponbare. **f** Roots in non-transgenic Nipponbare as a negative control. Bar = 2 mm

**Fig. 3:**
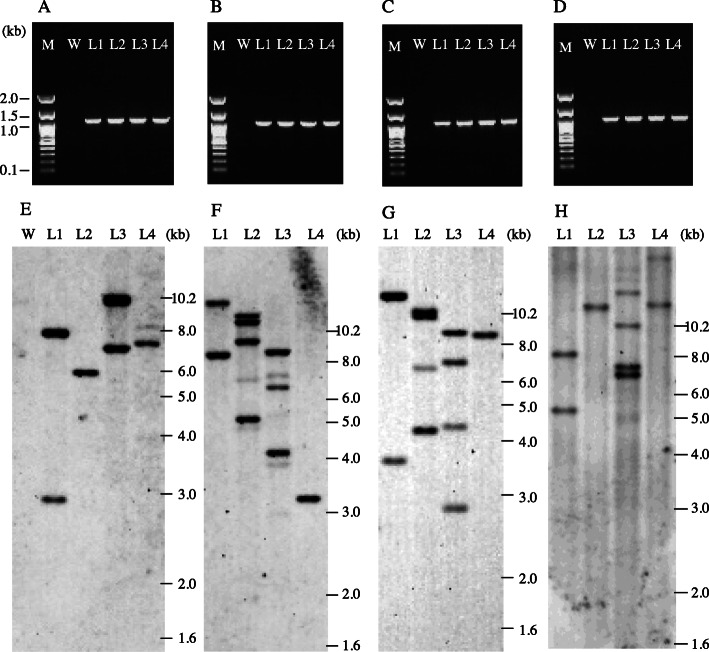
Detection of transgenes in four lines of T_0_ plants from 4 accessions. **a**, **e***O. barthii* W1467, **b**, **f***O. glumaepatula* W2184, **c**, **g***O. rufipogon* W1551, and **d**, **h***O. brachyantha* W1711. **a**–**d** PCR amplification of full-length *hpt* gene from genomic DNA prepared from each independent line of transgenic plants (T_0_). Lanes: M, molecular weight marker; W, non-transgenic control; L1–L4: 4 independent lines of each transgenic wild *Oryza* accession. **e**–**h** Southern blot analysis of transgenic T_0_ plants with *hpt* probe. Lanes: W, non-transgenic control; L1–L4, T_0_ plants of the same lines in (**a**–**d**)

Next, we tried to generate transgenic plants expressing GFP by introducing the same vector used in the previous analysis into *O. barthii* (2 accessions), *O. glumaepatula* (8), *O. rufipogon* (15), and *O. brachyantha* (1) (Table 6). The transformation of *O. brachyantha* W1711 is shown as an example (Fig. 1a–i). A GFP signal was observed at 10 days after infection (Fig. 1a). Plants were selected on callus-inducing medium with antibiotics (Fig. 1b) and regenerated (Fig. 1c). Regenerated shoots were transferred to rooting medium (Fig. 1d) and then to pots with soil (Fig. 1g) and were grown to maturity. The regenerated T_0_ plants expressed GFP in leaves (Fig. 1e, f) and roots (Fig. 2). We confirmed the introduction of the transgene in regenerated T_0_ plants of *O. barthii* W1467, *O. glumaepatula* W2184, *O. rufipogon* W1551, and *O. brachyantha* W1711 by PCR (Fig. 3a–d) and found that the transformation events were all independent, with 1 to 4 T-DNA insertion loci, on the basis of the number of bands in Southern blot analysis (Fig. 3e–h).

**Table 6:** Accessions used to generate transgenic plants expressing GFP

Species, genome type	Accessions
*O. barthii*, AA	W1467, W1702
*O. glumaepatula*, AA	W1477, W2160, W2165, W2173, W2184, W2192, W2201, W2203
*O. rufipogon*, AA	W0106, W0107, W0610, W0621, W0625, W0629, W1239, W1551, W1666, W1669, W1681, W1685, W1865, W2014, W2265
*O. brachyantha*, FF	W1711

We obtained five or more regenerated plants from all accessions tested (Table 6). All grew to maturity without any discernable morphological abnormalities. All plants were fertile and set T_1_ seeds by self-pollination. We confirmed the transmission and segregation of the transgene in T_1_ seeds as GFP fluorescence in grains (Fig. 1h, i). From the results of Southern blot analysis, we picked two T_1_ lines with the transgene at either one or two loci from *O. barthii* W1467, *O. glumaepatula* W2184, *O. rufipogon* W1551, and *O. brachyantha* W1711 and tested its inheritance by observing GFP fluorescence of T_1_ grains (Table 7). The ratios of GFP-positive and -negative grains all fitted the expected 3:1 or 15:1 ratio, confirming the transmission of the transgene in a Mendelian manner.

**Table 7:** Inheritance and segregation of the transgenes

Species	Line number^a^	*GFP* gene in T_1_ seeds		Segregation ratio (χ^2^, *P*)
		Positive	Negative	
*O. barthii*, W1467	L1	56	4	15:1 (0.02, 0.89)
	L2	23	7	3:1 (0.04, 0.83)
*O. glumaepatula*, W2184	L1	57	3	15:1 (0.16, 0.69)
	L4	22	8	3:1 (0.04, 0.83)
*O. rufipogon*, W1551	L1	56	4	15:1 (0.02, 0.89)
	L4	22	8	3:1 (0.04, 0.83)
*O. brachyantha*, W1711	L1	57	3	15:1 (0.16, 0.69)
	L2	23	7	3:1 (0.04, 0.83)

^a^Line number is the same as in Fig. 3

## Conclusion

Our results show that a wide range of wild *Oryza* accessions, including those distantly related to cultivated species, can be genetically transformed by *Agrobacterium* by using the immature embryo method (Hiei and Komari 2008). Modification of the method increased the number of accessions that regenerated. Further attention to medium composition and conditions of infection will broaden the range of wild *Oryza* accessions that can be transformed.

We generated transgenic plants from *O. rufipogon* accessions W0106 and W1681 by the immature embryo method but not by the conventional scutellum callus method (data not shown). This difference supports the superiority of the immature embryo method for the transformation of wild *Oryza* accessions. This method opens the door to genome editing, accelerating the study of wild *Oryza* genetic resources for molecular genetic analysis and future use in molecular breeding.

## Supplementary information

**Additional file 1: Supplemental Table 1.** Composition of media (1 L) used in this study. **Supplemental Table 2.** Work flow of test of regeneration from callus derived from immature embryos of wild Oryza species.

## Data Availability

All datasets are available from the corresponding author on reasonable request.

## Abbreviations

*hpt*: *hygromycin phosphotransferase*
*GFP*: *green fluorescent protein*
DAP: day after pollination
