# Supplementary material for: Agrobacterium-Mediated Genetic Transformation of Wild Oryza Species Using Immature Embryos
Source: Rice (N Y). 2020 Jun 3;13:33. doi: 10.1186/s12284-020-00394-4 (PMC7270233; doi:10.1186/s12284-020-00394-4)
Supplement: Supplementary file 1 — Additional file 1: Supplemental Table 1. Composition of media (1 L) used in this study. Supplemental Table 2. Work flow of test of regeneration from callus derived from immature embryos of wild Oryza species. [file 12284_2020_394_MOESM1_ESM.docx]

**Supplemental Table 1.** Composition of media (1 L) used in this study.

| Medium | Composition |
| --- | --- |
| nN6C | 100 mL of 10× N6 major salts (28.3 g/L KNO_3_, 4.63 g/L (NH_4_)_2_SO_4_, 1.66 g/L CaCl_2_·2H_2_O, 1.85 g/L MgSO_4_·7H_2_O, and 4.0 g/L KH_2_PO_4_), 10 mL of 100× FeEDTA (2.78 g/L FeSO_4_·7H_2_O, and 3.73 g/L Na_2_EDTA), 10 mL of 100× N6 minor salts (440 mg/L MnSO_4_·4H_2_O, 150 mg/L ZnSO_4_·7H_2_O, 160 mg/L H_3_BO_3_, and 80 mg/L KI),10 mL of 100× N6 vitamins (100 mg/L thiamine hydrochloride, 50 mg/L pyridoxine hydrochloride, 50 mg/L nicotinic acid, and 200 mg/L glycine), 10 mL of 100 mg/L 2,4-D, 5 mL of 100 mg/L NAA, 1 mL of 100 mg/L 6BA, 20 g sucrose, 55 g sorbitol, 0.5 g proline, 0.5 g vitamin assay casamino acids, 5 g gelrite, and 2 mL of 12.5 mg/mL meropenem trihydrate, pH 5.8 |
| N6R | 50 mL of 10× N6 major salts, 10 mL of 100× FeEDTA, 10 mL of 100× N6 minor salts, 100 mL of N6 vitamins, 500 µL of 1 mg/mL kinetin, 20 g sucrose, 30 g sorbitol, 1 g vitamin assay casamino acids, 100 mL of 10× AA amino acids (8.76 g/L glutamine, 2.66 g/L aspartic acids, 1.74 g/L arginine, and 75 mg/L glycine), and 4 g gelrite, pH 5.8 |
| N6F | 50 mL of 10× N6 major salts, 10 mL of 100× FeEDTA, 10 mL of 100 N6 minor salts, 10 mL of N6 vitamins, 15 g sucrose, 30 g sorbitol, 1 g vitamin assay casamino acids, 100 mL of 10× AA amino acids, and 3 g gelrite, pH 5.8 |
| CCMC | 100 mL of 10× CC major salts (12.12 g/L KNO_3_, 6.4 g/L NH_4_NO_3_, 5.88 g/L CaCl_2_·2H_2_O, 2.47 g/L MgSO_4_·7H_2_O, and 1.36 g/L KH_2_PO_4_), 10 mL 100× FeEDTA, 10 mL of 100× CC minor salts (1115 mg/L MnSO_4_·4H_2_O, 576 mg/L ZnSO_4_·7H_2_O, 2.5 mg/L CuSO_4_·5H_2_O, 24 mg/L Na_2_MoO_4_·2H_2_O, 2.8 mg/L CoSO_4_·7H_2_O, 310 mg/L of H_3_BO_3_, and 83 mg/L KI), 10 mL of 100× CC vitamins (9 g/L myoinositol, 850 mg/L thiamine hydrochloride, 100 mg/L pyridoxine hydrochloride, 600 mg/L nicotinic acid, and 200 mg/L glycine), 200 µL of 10 mg/mL 2,4-D, 2 mL of 100 mg/L NAA, 2 mL of 100 mg/L 6BA, 20 g maltose monohydrate, 36 g mannitol, 0.5 g proline, 0.5 g vitamin assay casamino acids, 5 g gelrite, and 2 mL of 12.5 mg/mL meropenem trihydrate, pH 5.8 |
| NBPRC | 100 mL of 10× N6 major salts, 10 mL of 100× FeEDTA, 10 mL of 100× B5 minor salts (1320 mg/L MnSO4·4H_2_O, 200 mg/L ZnSO_4_·7H_2_O, 2.5 mg/L CuSO_4_·5H_2_O, 25 mg/L Na_2_MoO_4_·2H_2_O, 2.5 mg/L CoCl_2_·6H_2_O, 300 mg/L H_3_BO_3_, and 75 mg/L KI), 10 mL of 100× B5 vitamins (10 g/L myoinositol, 1 g/L thiamine hydrochloride, 100 mg/L pyridoxine hydrochloride, and 100 mg/L nicotinic acid), 200 µL of 10 mg/mL 2,4-D, 10 mL of 100 mg/L NAA, 10 mL of 100 mg/L 6BA, 30 g maltose monohydrate, 0.5 g proline, 0.5 g vitamin assay casamino acids, 5 g gelrite, 0.1 mL 100 mM CuSO_4_, 10 mL of 30 g/L glutamine, pH 5.8 |
| RNM | 100 mL of 10× N6 major salts, 10 mL of 100× FeEDTA, 10 mL of 100× B5 minor salts, 10 mL of 100× B5 vitamins, 10 mL of 100 mg/L NAA, 10 mL of 100 mg/L NAA, 30 mL of 100 mg/L 6BA, 30 g maltose monohydrate, 0.3 g proline, 0.3 g vitamin assay casamino acids, 4 g agarose Type I, and 10 mL of 30 g/L glutamine, pH 5.8 |
| MSI | 50 mL of 10× MS major salts (19 g/L KNO_3_, 16.5 g/L NH_4_NO_3_, 4.4 g/L CaCl_2_·2H_2_O, 3.7 g/L MgSO_4_·7H_2_O, and 1.7 g/L KH_2_PO_4_), 10 mL of 100× FeEDTA, 10 mL of 100× MS minor salts (2230 mg/L MnSO_4_·4H_2_O, 1060 mg/L ZnSO_4_·7H_2_O, 2.5 mg/L CuSO_4_·5H_2_O, 25 mg/L Na_2_MoO_4_·2H_2_O, 2.5 mg/L CoCl_2_·6H_2_O, 620 mg/L H_3_BO_3_, and 83 mg/L KI), 10 mL of modified MS vitamins (10 g/L myoinositol, 100 mg/L thiamine hydrochloride, 50 mg/L pyridoxine hydrochloride, 50 mg/L nicotinic acid, and 200 mg/L L-glycine), 2 mL of 100 mg/L IBA, 15 g sucrose, 1 g vitamin assay casamino acids, 3 g gelrite, and 100 mL of 10× AA amino acids, pH 5.8 |

**Supplemental Table 2**. Work flow of test of regeneration from callus derived from immature embryos of wild *Oryza* species.

|  | A | B | C | D |
| --- | --- | --- | --- | --- |
| 1st callus induction (1 week) | nN6C | CCMC | nN6C | CCMC |
|  | ↓ | ↓ | ↓ | ↓ |
| 2nd callus induction (1 week) | nN6C | CCMC | nN6C | CCMC |
|  | ↓ | ↓ | ↓ | ↓ |
| Pre-regeneration (1 week) |  | NBPRC | NBPRC |  |
|  |  | ↓ | ↓ |  |
| Regeneration (2 weeks) | N6R | RNM | RNM | N6R |
|  | ↓ | ↓ | ↓ | ↓ |
| Rooting (2 weeks) | N6F | MSI | MSI | N6F |
